# IoT Smart Parking System Based on the Visual-Aided Smart Vehicle Presence Sensor: SPIN-V

**DOI:** 10.3390/s20051476

**Published:** 2020-03-08

**Authors:** Luis F. Luque-Vega, David A. Michel-Torres, Emmanuel Lopez-Neri, Miriam A. Carlos-Mancilla, Luis E. González-Jiménez

**Affiliations:** 1 Centro de Investigación, Innovación y Desarrollo Tecnológico CIIDETEC-UVM, Universidad del Valle de México, Tlaquepaque, Jalisco 45601, Mexico; davidadrian.michel@my.uvm.edu.mx (D.A.M.-T.); emmanuel.lopezne@uvmnet.edu (E.L.-N.); miriam.carlos@uvmnet.edu (M.A.C.-M.); 2 Department of Electronics Systems and Computing, ITESO AC, Tlaquepaque, Jalisco 45604, Mexico; luisgonzalez@iteso.mx

**Keywords:** smart city, smart vehicle presence sensor, smart parking system, sensing solution

## Abstract

Humanity is currently experiencing one of the short periods of transition thanks to novel sensing solutions for smart cities that bring the future to today. Overpopulation of cities demands the development of solid strategic plannings that uses infrastructure, innovation, and technology to adapt to rapid changes. To improve mobility in cities with a larger and larger vehicle fleet, a novel sensing solution that is the cornerstone of a smart parking system, the smart vehicular presence sensor (SPIN-V, in its Spanish abbreviation), is presented. The SPIN-V is composed of a small single-board computer, distance sensor, camera, LED indicator, buzzer, and battery and devoted to obtain the status of a parking space. This smart mobility project involves three main elements, namely the SPIN-V, a mobile application, and a monitoring center, working together to monitor, control, process, and display the parking space information in real-time to the drivers. In addition, the design and implementation of the three elements of the complete architecture are presented.

## 1. Introduction

Nowadays, several applications in scientific areas such as medicine, agriculture, social sciences, and computer sciences, as well as non-scientific areas, such as government, society, and industry, among others, have been boosted by the implementation of Internet of things (IoT) techniques. IoT is expressed in diverse areas that are classified according to the problems that can be solved, e.g., those in health, agriculture, networks, cities, and sports, among others. Future sensing solutions will be embedded in large automation systems, such as smart factories, buildings, and cities.

Some of the current solutions are focused on the implementation of smart cities solutions. Smart cities is a concept that allows integrating technologies into a community intending to make life easier with minimal effort. These are the result of the need to guide our lives toward sustainability. The initiatives of smart and sustainable cities do not have to be seen as a model of a distant and unattainable future, but as a necessity to the current reality to face the challenges of the current societies, where information and communication technologies (ICT) would play a crosscutting role as articulating tools that guarantee a better result and, where appropriate, facilitate social cohesion, security, and sustainability [1].

Smart cities use infrastructure, innovation, and technology for adapting the changes brought about by the overpopulation of cities and thus contribute to the reduction of carbon dioxide emissions, decreasing energy consumption, and promoting economic, social, and environmental development. One of the first steps to be implemented to upgrade the traditional cities to become smart cities is smart parking [2].

Many cities around the world have already started implementations of smart parking projects, making life easier. Intelligent parking helps drivers to efficiently and effectively search for parking spaces through information and communication technology [3]. In addition, intelligent parking spaces are systems that optimize the way of parking, either by streamlining the process or by reducing the space required. This proposal seeks to implement an attitude of respect towards the environment [4].

Many proposals are working on this approach. Some of them require using the global positioning system (GPS) to know the available locations (e.g., [5]). Other works focus on making reservations without ensuring the space availability (e.g., [6,7]). In this work, a new parking system is presented.

The proposed system is composed of three elements. The first is the development of an Intelligent Vehicular Presence Sensor (SPIN-V, in its Spanish abbreviation) installed in parking spaces, which stores, processes, and notifies the state of a parking space. The second is the intelligent parking system (SEI-UVM, in its Spanish abbreviation); it is available through a mobile application to monitor and manage parking spaces and also allows users to make reservations of any available space. The third is a monitoring center that gathers information about the complete system, users, and SPIN-V sensors. A case of study of the sensor functionality is presented and compared with the existing ones. In the next section, some of the most representatives proposals in this area are presented.

## 2. Related Works

In this section, some of the most representatives proposal in this area are described.

In [8], an architecture for smart parking is proposed employing a mobile application connected to the cloud. The system provides parking facilities in real-time and users are able to reserve places and make payments before arriving at the parking space. There exist other systems that use wireless communication to reserve places and recommendations of nearest parking spaces through the global positioning system (GPS) [5]. The system transmits the availability of spaces every 2 min. If all parking spaces are occupied, no actions are considered; in the other case, any user is able to reserve a place within 2 km of their location. The GPS coordinates are available for any smartphone; then, the user receives a message with directions. The application requires a WiFi connection and no action is performed if there is a car parked in every parking spot.

An intelligent parking algorithm is presented in [6], which calculates the optimal parking space for the user based on the distance of trajectory and time. It should be noted that the system does not have the reservation service and is subject to the availability of the space at that time. On the other hand, in the smart parking system proposed in [7], the authors presented the management of parking spaces in real-time using the cloud as a means of communication and a database. The system has a mobile application to make space reservations and an ultrasonic sensor placed on the ground connected via Ethernet.

A novel parking system designed for smart cities is proposed in [9]. The system is connected to the cloud, and the guidance function is based on the Ant Colony Optimization algorithm (ACO) to calculate the shortest path between two points, the user and the available space. The authors did not present any application. There exist some proposals which focus their efforts on the available technologies for smart parking spaces. In [10], sensors, technologies, and interfaces are used to collect and display real-time parking occupancy information. Some applications are compared with this proposal and the results show that the parking occupancy sometimes cannot be displayed due too few connected users to the application. The authors explained that all the existing smart parking technologies and applications are not suitable for open parking lots due to varying environmental conditions and high expenditure.

These current smart parking systems allow parking a vehicle quickly and efficiently. However, they require special infrastructure that is expensive in terms of installation, time, and money. The preceding raises an area of innovation opportunity in the automation of private parking lots looking for practicality in its installation, modularity, and design, which may trigger the introduction of new business models.

## 3. National Observatory of Smart Environments OBNiSE

Research centers, universities, and industries have gathered efforts to collaborate on technological developments and projects. The focus of this proposal is educational institutions. Universities have undertaken projects with industry and government.

This section presents the first element of the proposal, the monitoring center located at the largest university in Mexico. The Universidad del Valle de México (UVM) [11] is the largest private university in Mexico, which considers research as one of the keys of education; with over 30 campuses around Mexico; 179,073 high school, master and university students; 8726 teachers; more than 60 researchers; 200,000 graduate students; and 5974 administration staff (UVM, 2018), it was responsible for the creation of the Research Center of Investigation, Innovation and Technological Development (CIIDETEC-UVM Centro de Investigación, Innovación y Desarrollo Tecnológico (UVM) in Spanish) in 2009.

CIIDETEC-UVM as a research center has the *National Observatory of Intelligent Environments (OBNiSE)* from where various investigations and projects are carried out. The objective of OBNiSE is integrating strategies, methods, methodologies, prototypes, and, in turn, monitoring the behavior of new systems and devices connected to an internal network to provide an integrated system and services. The observatory is the place where the SEI-UVM system is installed and it allows the monitoring, control, and supervision of the functionality, creation, and development of the system. The explanation of the system is presented in the next section.

## 4. Smart Parking System Prototype

### 4.1. Smart Parking System SEI-UVM

**Figure 1:**
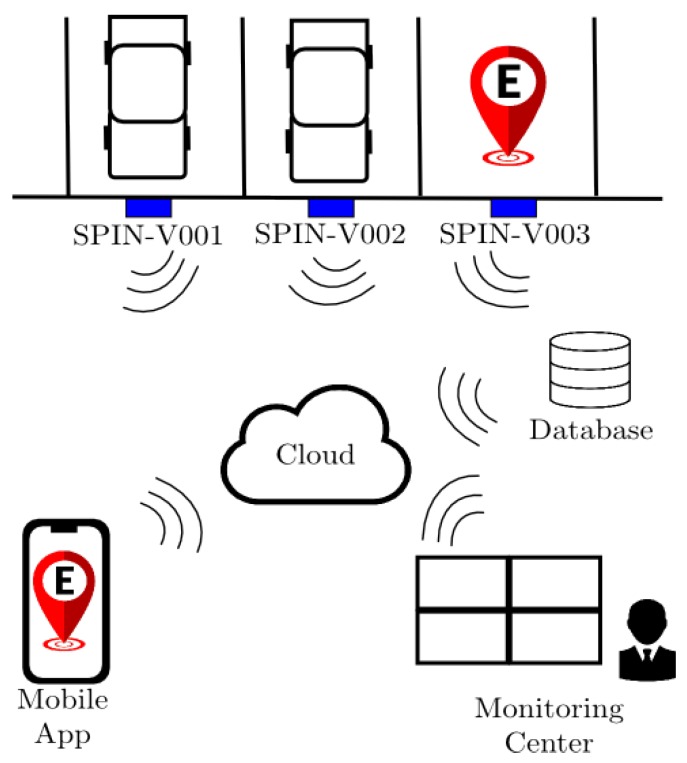
Smart parking system SEI-UVM.

The proposed SEI-UVM is a smart parking system that is devoted to private parking lots and composed of three main elements: *the SPIN-V, a mobile application*, and a *monitoring center* (see Figure 1). Each parking space is equipped with a SPIN-V located in the middle of the backend of parking spaces. The mobile application is available for the user/driver to control and reserve a parking space while the monitoring center can be operated by the owner of the private parking lots to manage and control the parking spaces and the reservations. The information shared by the app is stored in a database and observed by the monitoring center at OBNiSE. All the information is sent from the SPIN-V to the cloud. In addition, the exchange of information is available through WiFi protocol communication.

### 4.2. Network Architecture of the Smart Parking System SEI-UVM

The SEI-UVM can be used as a management and control system of private parking lots, in which the availability of parking spaces are shown in real-time to the driver/user through a mobile app. In this app, the user is able to enter a profile and register the vehicle’s license plate, monitor the available parking spaces up-to-the-minute, reserve a parking space, and obtain different route recommendations to get to the parking lot. On the other hand, the SPIN-V involves a distance sensor to detect the entry of a vehicle to the parking space, a camera to take an image of the vehicle’s plate, and an LED indicator and buzzer to inform the status of the parking space. Moreover, the information on the occupation of the parking spaces is displayed in real time to the monitoring center that ensures 24/7 functionality.

**Figure 2:**
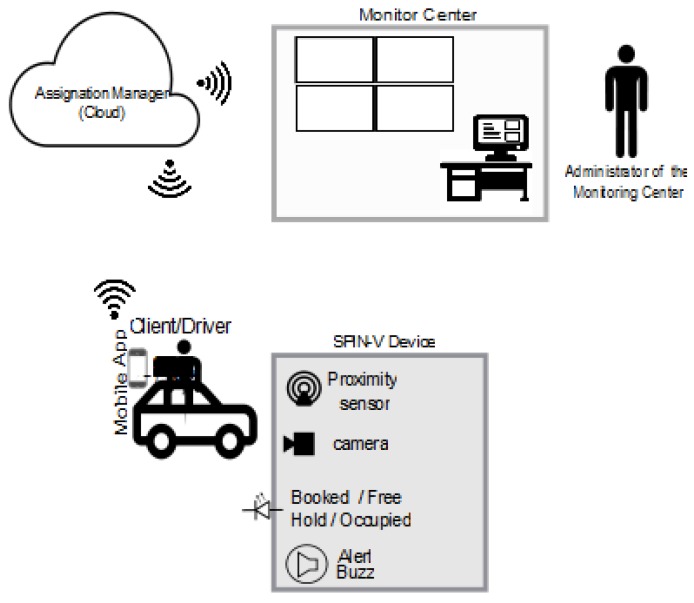
Network architecture of the SEI-UVM.

The proposed network architecture of the smart parking system SEI-UVM is depicted in Figure 2. Moreover, a more detailed explanation about the performance of the complete smart parking system is carried out below.

When a new vehicle arrives and is sensed by the proximity distance sensor of the SPIN-V, it takes an image of the vehicle’s plate and performs an image processing algorithm to convert from image to text. Then, the plate’s number is sent to the assignation manager at the monitoring center. The description of the process and an example explanation are presented in Section 5.

**Figure 3:**
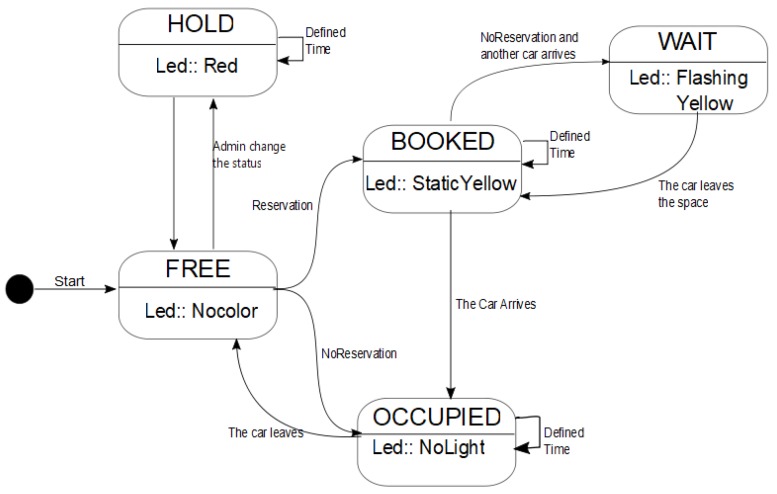
State diagram of the SPIN-V sensor at parking lots.

A parking space can be in one of the following statuses: FREE, OCCUPIED, BOOKED, HOLD, and WAIT. The app shows users what status each parking space is inside the parking lot. Each status has a defined LED color assignation. Figure 3 shows a state diagram and its corresponding transitions.

FREE$\overset{}{\to }$Nocolor, OCCUPIED$\overset{}{\to }$NoLight, BOOKED$\overset{}{\to }$Yellow, HOLD$\overset{}{\to }$Red, WAIT$\overset{}{\to }$FlashingYellow.

The initial state of a parking space is FREE. If the driver/user has the app on his/her phone or they are willing to download it to use the SEI-UVM, he/she will receive the parking availability in real-time. Then, the user/driver can decide between booking a parking space through the app or looking for a parking space traditionally. If the driver/user book a parking space by the app, the chosen parking space status changes to BOOKED. He/she has to travel to the location of the parking space. When he/she arrives with his/her car to the parking space, the status of the SPIN-V changes to OCCUPIED and, when the car leaves, the state changes to FREE. When a difficulty is presented such as another driver enters a parking space with status BOOKED, the SPIN-V sensor will take some actions to always ensure the availability of the system.

**Figure 4:**
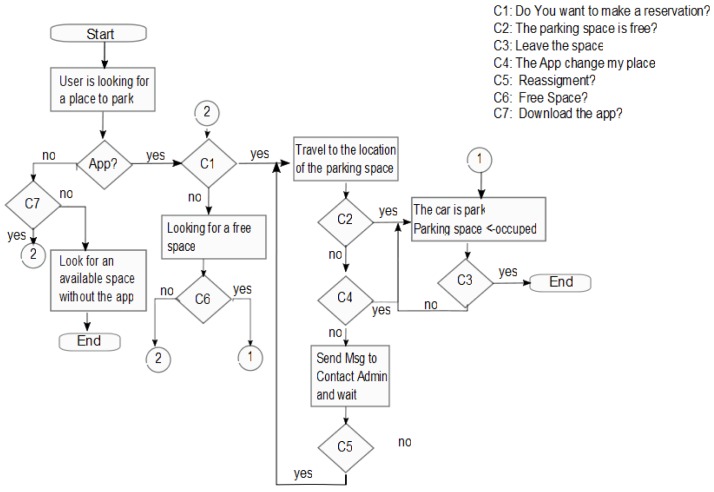
Flow diagrams that represent the user/driver process.

The general process and the actions considered when a driver/user is looking for a parking space is depicted in Figure 4. Whether a user looks for a space to park, two scenarios can be possible. (*1) The user has the application.* (*2)The user does not have the application.* If the user already has the app, then looks for an available space, the user can find one, is able to reserve the space, arrive at the space, and then leave the space FREE. (2) If a user does not have the app, then the user is able to download the app or look for a space traditionally. The complete process with exceptions is shown in Figure 4.

This work considers the three main scenarios presented above, during or after the interaction with the mobile application. It is considered that a user/driver only can make a reservation when a space state is FREE. The cases are details below.

**Figure 5:**
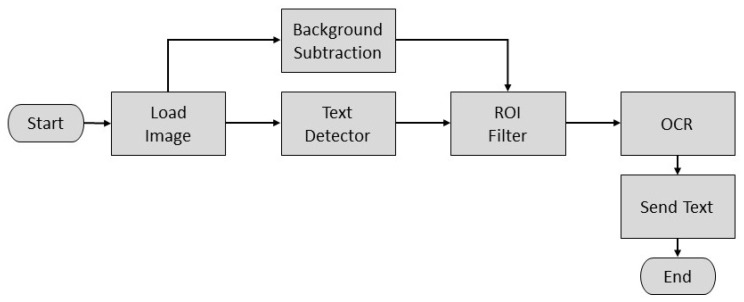
Conversion from image to text.

**Figure 6:**
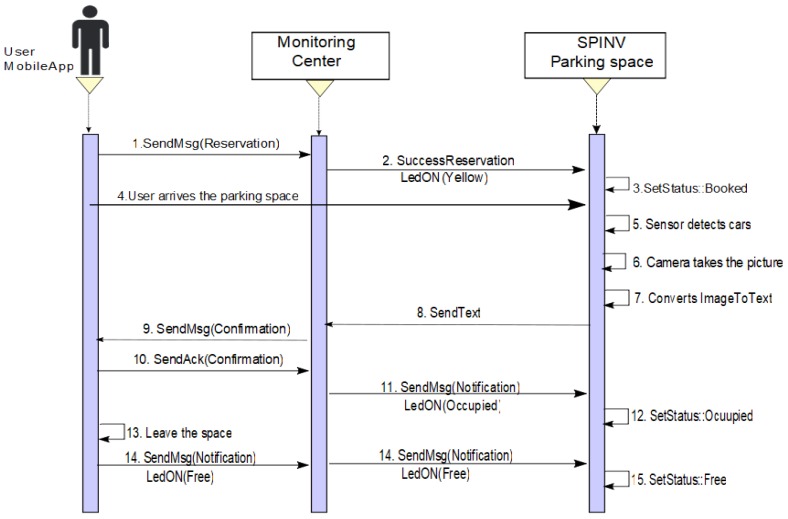
Sequence diagram of Scenario 1. Reserve/arrive to the booked parking space.

**Figure 7:**
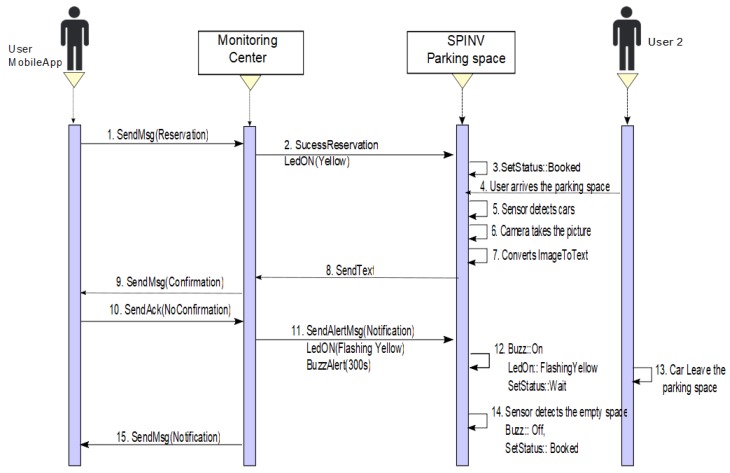
Sequence diagram of Scenario 2a. Reserve/someone else park in the space and the car leaves the space.

**Figure 8:**
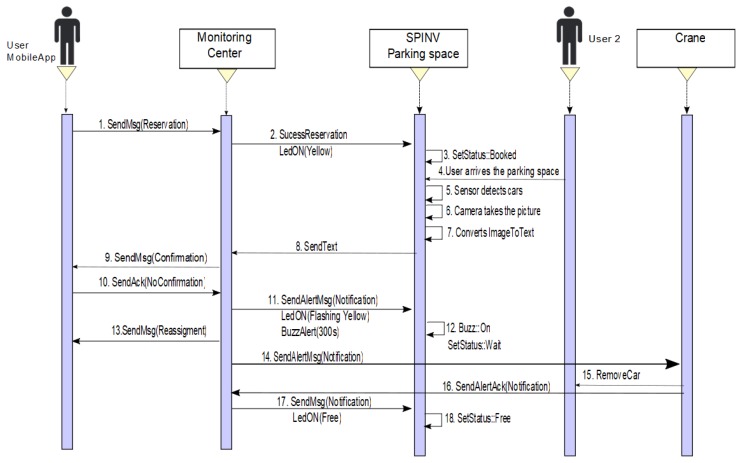
Sequence diagram of Scenario 2b. Reserve/someone else park in the space and the car does not leave the space.

**Figure 9:**
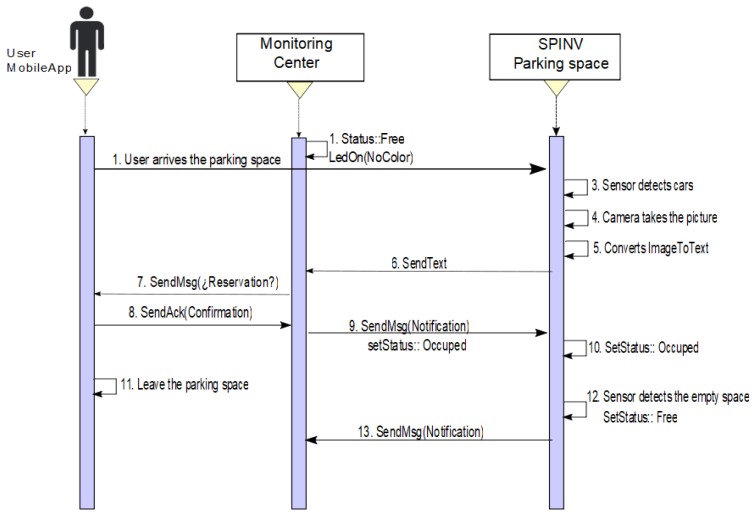
Sequence diagram of Scenario 3a. There is no reservation, a car arrives to the space, and the user/driver has the app.

**Figure 10:**
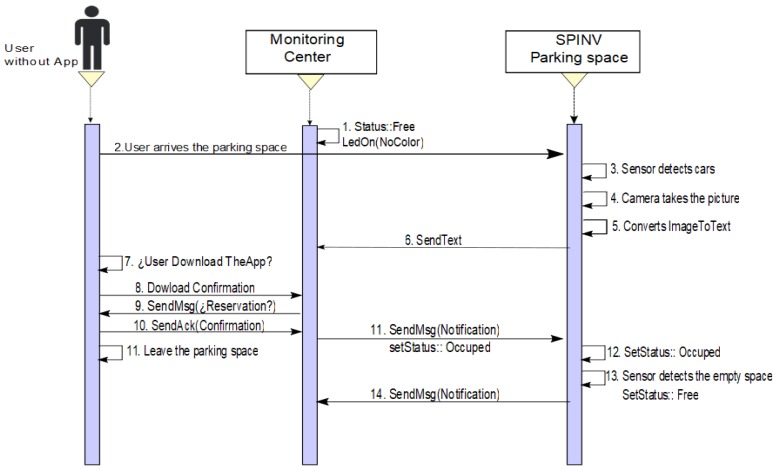
Sequence diagram of Scenario 3b. There is no reservation, a car arrives to the space, and the user/driver does not have the app.

- **Scenario 1: Make a reservation/park in the booked parking space**. The driver/user sets a reservation via the app/ Through the app, a requirement to the assignation manager in the monitoring center is sent. The SPIN-V LED (SpinV:LEDInd) of the reserved parking space is set to yellow. When the SPIN-V distance sensor (SpinV:Sensor) detects the arrival of a new vehicle, it takes the vehicle plate image with the SPIN-V Camera (SpinV:Camera), and internally gets the text from the image vehicle plate (see Figure 5), which is sent to the assignation manager and stored. The cloud informs the monitor service of the new parked vehicle and validates if the image plate is equal to the reservation, confirming to the reservation owner via app if it is correct, and the user confirms its arrival. Finally, the SPIN-V LED is set off and the state of the parking lot is set to OCCUPIED. The parking space remains in this state until the user leaves, when the state of the parking lot changes to FREE state until a new reservation or arrival is executed. Figure 6 depicts the sequential diagram of the process in Scenario 1.
- **Scenario 2: Make a reservation/someone else parked in the booked parking space.** The scenario two is divided into two situations. One of them is when someone else parked in the space but leaves the space. The other is someone else parked in the space and the car has not left the space. In both cases, the user/driver makes a reservation via the app and sends the requirement to the assignation manager. The SpinV:LEDInd of the reserved parking space is set to yellow, thus, when the SpinV:Sensor detects a new vehicle, it takes the vehicle plate image with the SpinV:Camera, and internally gets the text of the image vehicle plate, which is sent to the assignation manager and stored. The cloud informs the monitor service of this new parked vehicle and validates if the image plate is equal to the reservation; if it is not confirmed, the monitoring center sends a message alert to the SPIN-V and the SpinV:LEDInd is set to flashing yellow and the SPIN-V buzzer (SpinV:Buzz) sounds. Then, the following can happen.
  − **Scenario 2a. The car leaves the space**. The status of the parking space is set to OCCUPIED, the SpinV:Buzz sounds for 5 min or until the car leaves the space. If the car leaves the space, the parking space state returns to BOOKED, SpinV:Buzz off, and SpinV:LEDInd is set to yellow, until the reserved parking space is set to FREE by the main user. Figure 7 presents the process.
  − **Scenario 2b. The car does not leave the space**. The status of the parking space is set to OCCUPIED, the SpinV:Buzz sounds for 5 min. If the car does not leave the space, the monitoring center has an agreement with a towing service to which it will send an alert to remove the intruder car. In the parking space, there will be an announcement with the towing services for the driver. The driver who makes the reservation is reassigned to another parking space. Finally, the state is set to WAIT for a defined time, after which it changes to FREE. Figure 8 presents the process.
- **Scenario 3: No reserve/park in the parking space.** This scenario is divided into two possible cases. One is when the user does not make a reservation but has the app. The other is when the user does not make a reservation and does not have the app. In both cases, if the parking space is FREE, SpinV:LEDInd is off, and the driver/user arrives at the parking space. The SpinV:Sensor detects the vehicle, the SpinV:Camera takes the vehicle’s plate image, changes the image to text, and sends the text to the assignation manager to be stored. The cloud informs the monitor service of this new parked vehicle. The next actions are decided according to the scenarios explained below.
  − **Scenario 3a. No reserve/the driver has the app**. If the user has the app, then the monitoring center, more specifically the assignation manager, will send the user a notification asking if he want to reserve the space, and then the SPIN-V status changes to OCCUPIED until the user leaves the space, when it changes its status to FREE. Figure 9 presents the process.
  − **Scenario 3b. No teserve/the driver does not have the app**. If the user does not have the app, the user will find a sign with the app information to download and make the reservation. If the user downloads the app, he/she can follow Scenario 3a. In the other case, if the user will be in the parking space for a short time and decides not to download the app, then the user should ask the parking administrator at the establishment for a parking ticket or provisional permit. The assignation manager then should be notified to change the status to OCCUPIED. Figure 10 presents the process.

Despite the scenario, when the Spin-V sensor detects that the vehicle is leaving the parking space, the SpinV:Camera takes the vehicle plate image, and then the plate vehicle text is sent to the cloud, which informs the monitor of the leaving vehicle to change the SPIN-V to status FREE.

**Figure 11:**
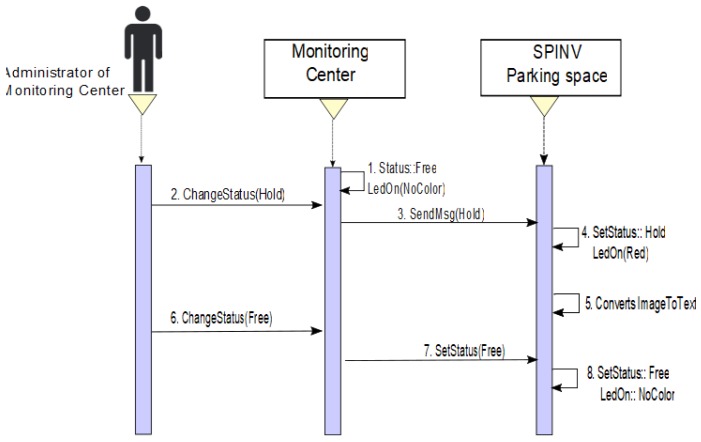
Sequence diagram when an administrator changes the status of one or more spaces to make an adjustment to sensors.

There is an exception when an administrator to the monitoring center needs to make some changes, maintaining or reconfiguring one or more spaces. In these case, it can directly change the status of the space(s) from the monitoring center, the SpinV:LEDInd is set to red, and the SPIN-V status changes to HOLD (see Figure 11).

All the sequence diagrams show the object interactions arranged in time, including the objects and classes involved in the scenarios and the sequence of messages exchanged between the objects needed to carry out the functionality of the three scenarios. These diagrams can be associated with the use case realizations in the logical view of the proposed system.

### 4.3. Design and Implementation of the IoT Sensor SPIN-V

The proposed system considers the design and development of the IoT based sensor SPIN-V, which is the mainstay of the smart parking system (SEI-UVM) and represents the first multi-disciplinary project carried out at the National Observatory of Smart Environments.

**Figure 12:**
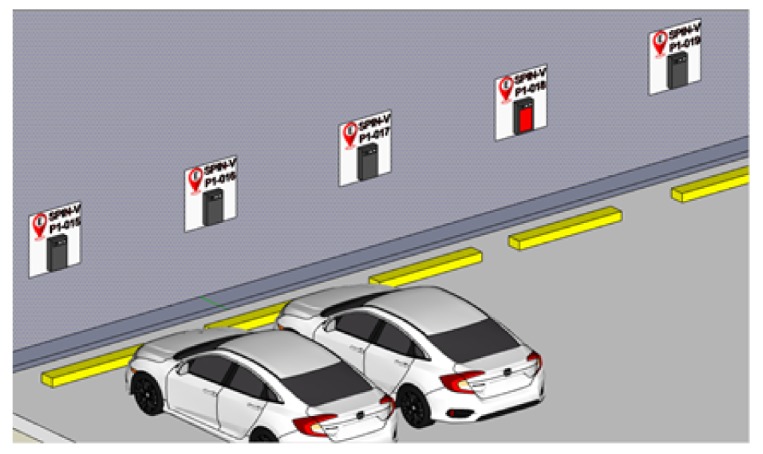
SPIN-V installed in the back end of the parking space for either commercial or residential use.

According to the logical view of the smart parking system SEI-UVM, the design considerations for the SPIN-V IoT sensor should contemplate the modularity, as well as the reduction in time and economic investment in the installation and maintenance, which give the IoT sensor the flexibility to offer several business models that could be applied for commercial and residential uses (see Figure 12).

#### 4.3.1. Design of the IoT Sensor SPIN-V

The SPIN-V is designed to be implemented with the following components: the Raspberry Pi 3 electronic card, an HC-SR04 ultrasonic sensor, the Pi v2 camera, a LED strip, a buzzer, and a battery. The description of each of the components is shown below.

- The Raspberry Pi is a small computer that consists of a motherboard on which a processor, graphics chip, and RAM is mounted. It is responsible for controlling the sensors, the led indicator, and the Pi Camera, as well as managing the communication between the overall system and the cloud services, so that the system can read and write data on them.
- An ultrasonic sensor HC-SR04 allows us to measure distances thanks to its two transducers, a piezoelectric emitter and a receiver. The emitter emits pulses of ultrasound at a frequency of 40 KHz; if an object is found, the sound waves will bounce. The amount of time it takes for the wave to return determines the distance. The sensor is used to sense the presence of a vehicle in a parking space.
- A Pi V2 camera, which offers high-speed, high-sensitivity images, is used to identify automobile plates.
- A LED strip is used in the physical system of the SPIN-V to indicate to the driver the status of the parking space. Within the secondary elements of the SPIN-V is the battery that is the power source for the Raspberry Pi.
- A buzzer is an electroacoustic transducer that produces a continuous sound or buzzing of the same tone (usually acute) and it serves as a signaling or warning mechanism.
- A battery electrochemical cell that transforms chemical energy into electricity powers all the components.

**Figure 13:**
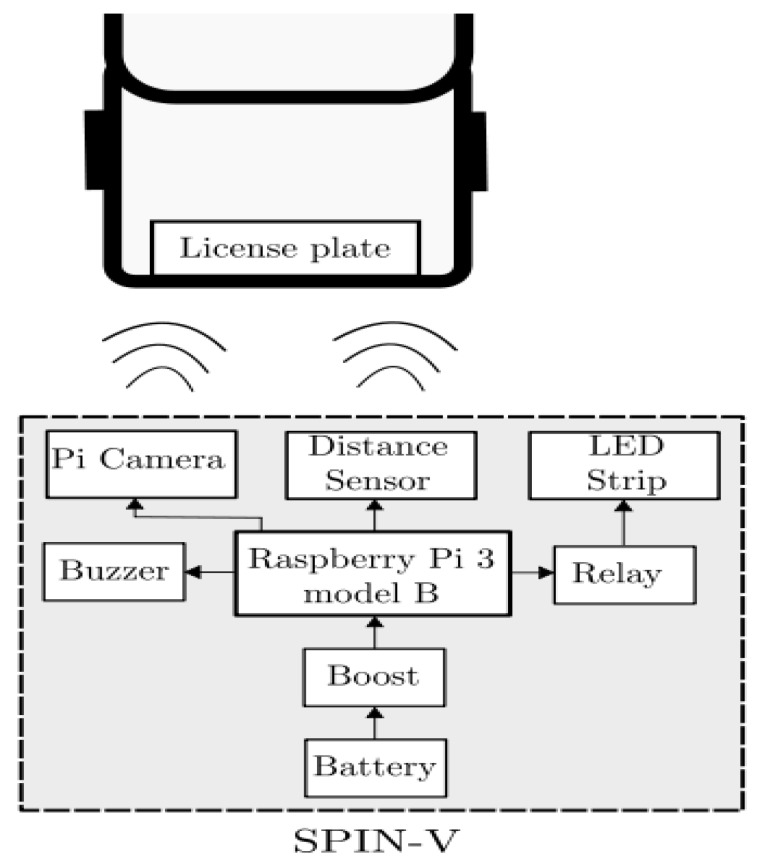
Connection diagram SPIN-V.

The connection diagram of the components of the SPIN-V is shown in Figure 13.

#### 4.3.2. Design of the Vision and Proximity System for the IoT Sensor SPIN-V

Vision systems are based on digital sensors within industrial cameras with optics specialized in acquiring images, so that hardware and software can process, analyze, and measure different characteristics to make decisions.

The Pi V2 camera is capable of performing automatic control functions with high-performance OmniBSI technology (high sensitivity, low cross talk, and low noise). As mentioned above, it is used to take the license plates of the car, when the presence of a car is detected in the parking space.

To define the best position and orientation of the Pi camera, it is necessary to take into account the following concepts in order to get the best the field of view of the camera. The fundamental parameters for the imaging system are as follows [12]:

- Field of Vision (FOV) is the visible area of an object.
- Working Distance (WD) is the distance from the front of the lenses to the object.
- Resolution is the minimum size of the characteristic of the object.
- Depth of Field (PC) is the maximum depth of the object that can be kept fully focused.
- Sensor Size is the size of the area of a camera sensor, normally specified in the horizontal dimension.

To obtain the field of view of the camera, it is necessary to define the designed parameter of the working distance WD =1 m and perform calculations based on the following specifications of the Pi v2 camera:

- Vertical field of view $\overset{}{\to }$ vAFOV $=48.8^{{}^{\circ}}$.
- Horizontal field of view $\overset{}{\to }$ hAFOV $=62.2^{{}^{\circ}}$.
- Camera resolution: 3280×2464 px.

**Figure 14:**
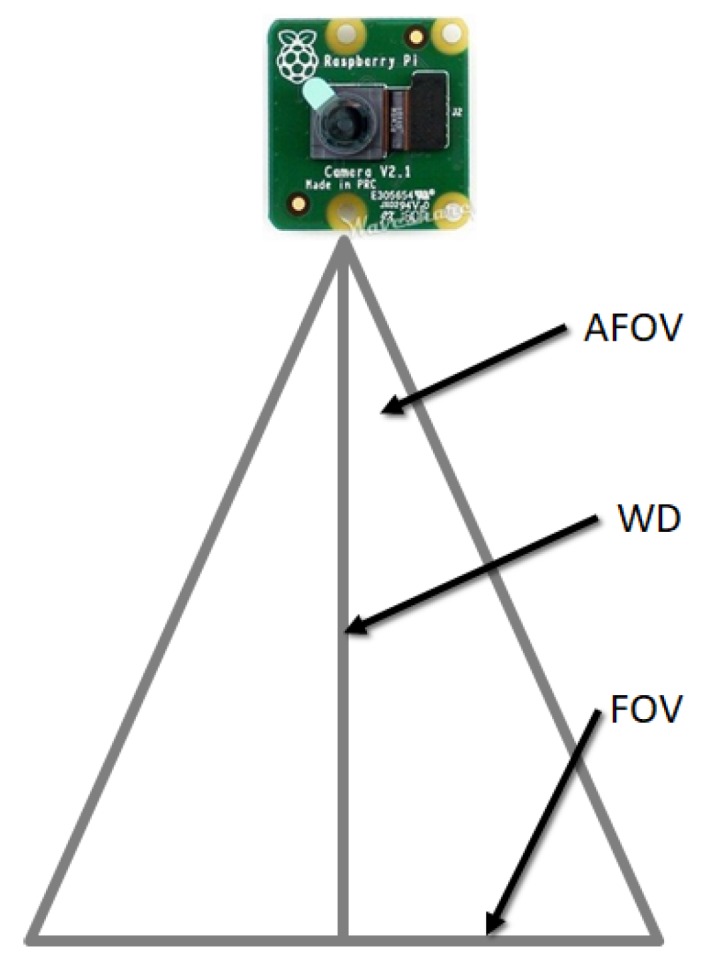
FOV Camera Pi V2.

The angular field of view (AFOV) is defined in degrees. The calculations of the image field of vision using the vAFOV of the Pi camera (Figure 14) are as follows:

(1) $$\tan\left(0.5\left(\mathrm{vAFOV}\right)\right)=0.5\frac{\mathrm{vFOV}}{\mathrm{WD}}$$

Clearing vFOV and replacing the premises, we have

(2) $$\mathrm{vFOV}=\frac{100}{0.5}\tan\left(0.5\left(48.8^{{}^{\circ}}\right)\right)=90.7\mathrm{cm}$$

Now, we do the same thing using the hAFOV and obtain

(3) $$\mathrm{hFOV}=\frac{100}{0.5}\tan\left(0.5\left(62.2^{{}^{\circ}}\right)\right)=120.6\mathrm{cm}$$

Calculating the resolution in pixels per meter (PPM), we have

(4) $$\mathrm{hPPM}=\frac{\mathrm{hPixeles}}{\mathrm{hFOV}}=\frac{3280\mathrm{px}}{1.206m}≃2719\mathrm{px}/m$$

(5) $$\mathrm{vPPM}=\frac{\mathrm{vPixeles}}{\mathrm{vFOV}}=\frac{2464\mathrm{px}}{0.907m}≃2716\mathrm{px}/m$$

To know the ideal location of the ultrasonic sensor, the field of vision calculations were carried out in the same way as in Equation (1) to Equation (5) considering the sensor specifications:

- Vertical field of view $\overset{}{\to }$ vAFOV${}_{SD}$$=15^{{}^{\circ}}$.
- Horizontal field of view $\overset{}{\to }$ hAFOV${}_{SD}$$=15^{{}^{\circ}}$.

The following results are obtained:

$${\mathrm{vFOV}}_{SD}=26.33\;\mathrm{cm}$$

$${\mathrm{hFOV}}_{SD}=26.33\;\mathrm{cm}$$

**Figure 15:**
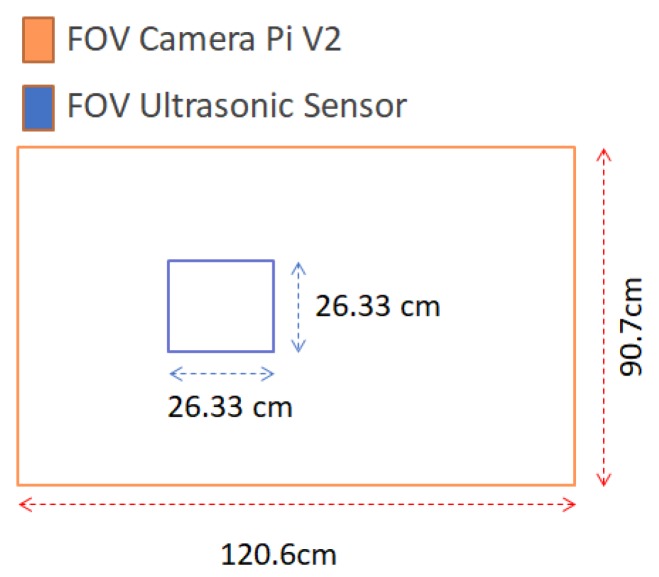
Field of view of both sensors.

Then, the field of view of both sensors is shown in Figure 15.

## 5. Results

### Implementation of the Vision System for the IoT Sensor SPIN-V

**Figure 16:**
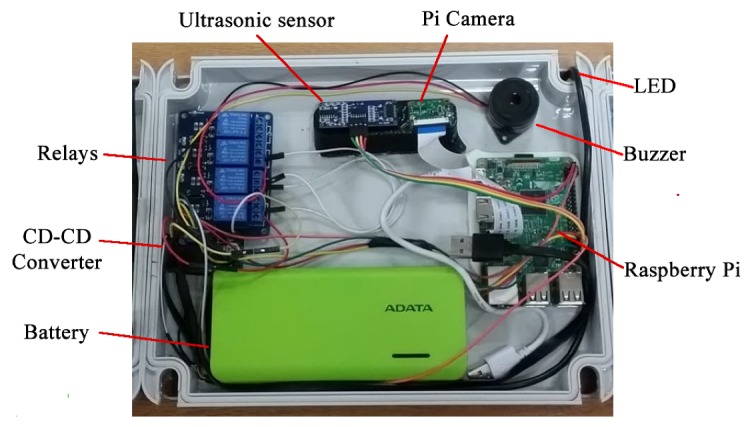
SPIN-V IoT Sensor prototype.

Once all components of the SPIN-V were defined, the bases for the raspberry pi, the camera pi, and the ultrasonic sensor were designed using SolidWorks and 3D printed. It is worth mentioning that the sensor’s base mount was designed based on the previous inclination calculations. Once the electronic integration was carried out, the prototype of the SPIN-V was obtained (see Figure 16).

**Figure 17:**
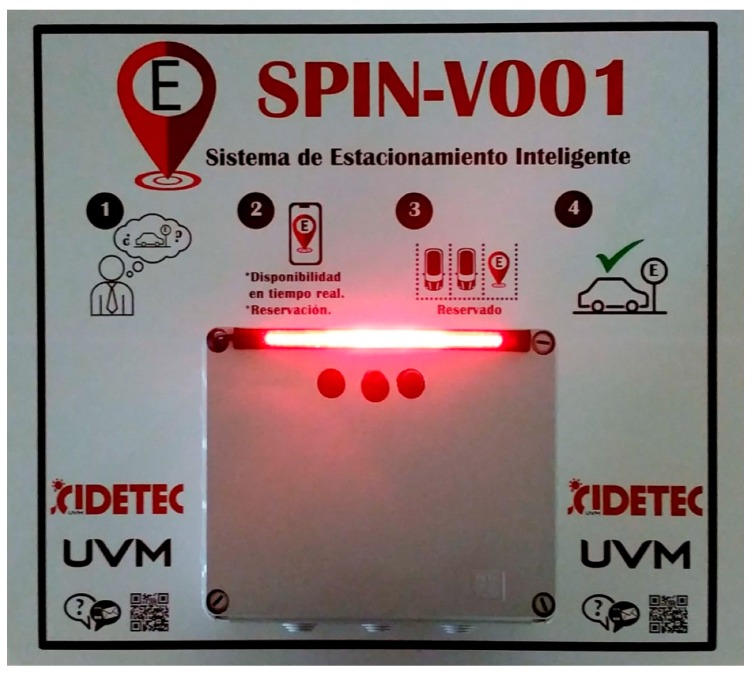
SPIN-V sensor mounted in the back end of the parking space.

To evaluate the performance of the SPIN-V, it was mounted in the back end of a parking space at the Universidad del Valle de México campus Guadalajara Sur (Figure 17).

**Figure 18:**
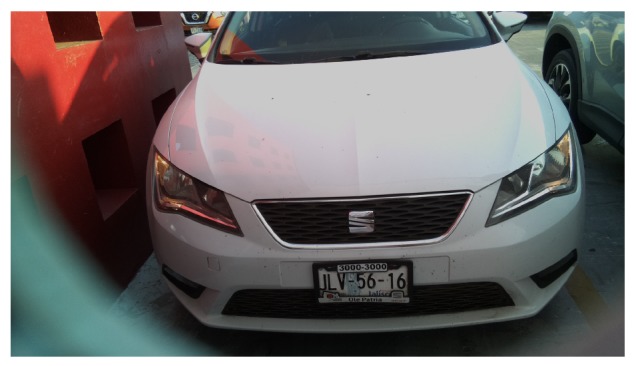
Image captured with the SPIN-V.

Once that SPIN-V was mounted at the height established by the design parameters, an image was obtained, as shown in Figure 18.

The firmware of the SPIN-V was programmed in Python, using the Python 3 Idle, which is installed at the factory with the Raspian Operating System on the Raspberry Pi 3. Several programming languages can be used on the Raspberry Pi. The Python language was chosen for the following reasons: it is simple, fast, flexible, ordered, portable, and with a variety of libraries included in the code.

For the license plate recognition, the image was processed to retrieve a string of characters corresponding to the license plate. First, the image was captured and loaded. Then, two operations were performed in the original image, a background subtraction and text detection.

The former permits the algorithm the segmentation of the car by defining and discarding the background. This was done as in [13] by defining a model for the background $I_{bg}\left(x,y\right)$, which is an image captured when there was no moving objects on the scene. Then, a binary foreground mask $I_{fg}\left(x,y\right)$ was calculated as a subtraction between the current image I(x,y) and the background model $I_{bg}\left(x,y\right)$ and a threshold operation as follows

(6) $$I_{fg}\left(x,y\right)=\left\{\begin{array}{ll} 255 & \mathrm{if}\;|I\left(x,y\right)-I_{bg}\left(x,y\right)|\geq th\;\\ 0 & \mathrm{otherwise}. \end{array}\right.$$

The threshold value th is defined to eliminate high frequency noise in the resulting image generated by variations in the light conditions of the scene.

The latter permits detecting zones in the original image that contains text-like patterns, which are defined as Regions of Interest (ROI). It was implemented based on the EAST (An Efficient and Accurate Scene Text) [14] detector, which is a highly accurate deep learning text detector in natural scene images. Its implementation in the OpenCV library [15] consists of a pre-trained deep learning model that receives the original image and generates a list of position and orientation for all the text-like regions detected in the image. For a detailed explanation of this model implementation, please refer to [16]. As it is common to encounter several texts-like zones on the back end of an automobile, a filtering stage must be implemented to select the ROI of interest and discard the others. Once the ROI is stored using the text detector and background subtraction stages, it can be located based on the size and position of the detected ROIs relative to the segmented car.

**Figure 19:**
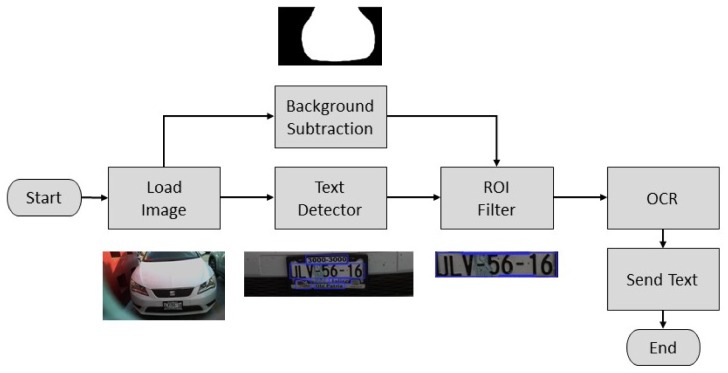
Diagram of the license plate recognition algorithm.

After that, the retrieved ROI is used as the input of an Optical Character Recognition (OCR) algorithm implemented by the Tesseract API [17]. This API is an open-source deep-learning OCR engine. It was originally developed at Hewlett Packard and is currently developed and maintained by Google. Finally, the output of the OCR function is defined as the string of characters of the license plate. The overall process is depicted in the diagram of Figure 19 and it is composed of standard digital image and computer vision functions which are included in the OpenCV library [15]. The algorithm was also implemented on the Raspberry Pi computer of the proposed system.

**Figure 20:**
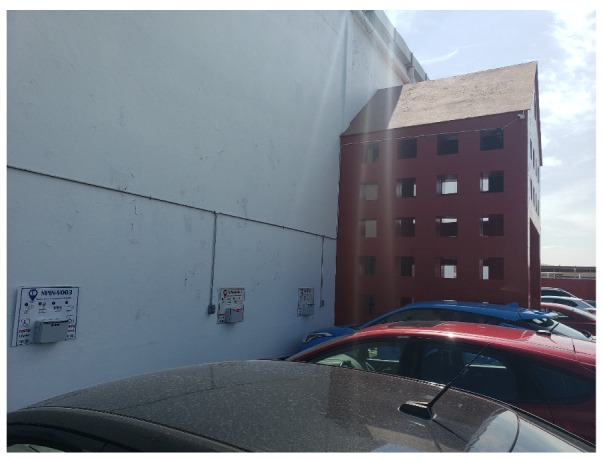
Three SPIN-V Installed in UVM.

**Figure 21:**
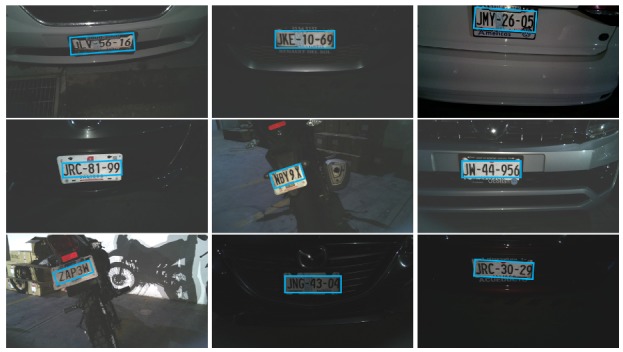
License plate identification results for different environmental conditions.

The complete system was evaluated using the mobile app, the SPIN-V, and the monitoring center. Moreover, three SPIN-V were mounted at the university to test the performance of the complete architecture (see Figure 20). The performance of the overall system was evaluated, obtaining satisfactory results. In this regard, Figure 21 shows the license plate identification results for different types of vehicles during different environment conditions. The images were captured using the SPIN-V sensor mounted at the university; hence, they only show automobiles and motorcycles. It can be noted that, despite drastic changes in environment illumination, size, position, and orientation of the plate, the identification was carried out correctly. Moreover, the response time of the overall solution was evaluated, resulting in an average of 6 s for execution of the whole system’s functionality including the following stages: the database reading and writing, sensor measurement and conditioning, image capturing and processing, and user’s mobile app updating.

## 6. Discussion

This novel sensing solution of the SPIN-V as a cornerstone of the SEI-UVM has been designed and developed as a compact device and adaptable to different environments since it can be used without the need for an expensive infrastructure installation, unlike the solutions of the current guided systems. The three main elements of the SEI-UVM working together in the proposed architecture open the possibilities to bring business, product development, marketing, and sales together, improving innovative product management.

It should be noted that the sensing solution of the SPIN-V was made with the integration of sensors in the market; the energy consumption is not optimized in this version and it will be added in future work. More advanced technologies in terms of power consumption have to be taken into account for the second version of the SPIN-V. Besides, a service provider for the Internet of Things (IoT) such as Sigfox should be considered to connect the SPIN-V with the digital universe.

Furthermore, some issues related to the effectiveness of the license plate identification algorithm should be improved. For instance, the sensitivity of the proposed solution to environmental light conditions must be considered throughout the implementation of the algorithm, as well as during the positioning of the camera and the overall system. Situations such as occlusions or direct exposure to sunlight must be handled properly by the system. On the other hand, despite the robustness of the text detection algorithm, high perspective distortion must be avoided as it can prevent the system from the text detection.

## 7. Materials and Methods

The manuscript describes the three main elements of the SEI-UVM. The process to build a SPIN-V could be followed by a reader with an engineering background in terms of hardware. Regarding the software, the code is intuitive and the program in Python is provided. Moreover, the mobile application has been developed in Android Studio and the .apk file is also provided. In this paper, the web application in which the monitor center displays the parking spaces in real-time is not shown because the graphical user interface is still in progress.

## 8. Conclusions

An integral solution of intelligent mobility is presented, which was implemented in a university campus outdoor car parking, aiming to reduce the search time for outdoor parking in times of greater road congestion within the campus.

The proposed architecture of the solution of the SEI-UVM is divided into three main blocks: SPIN-V, the mobile app, and the monitoring center (OBNiSE), where the key piece is the SPIN-V IoT sensor. It is designed as a compact device that can be adaptable to different environments and does not need an expensive infrastructure installation. In addition, it allows solving the challenges of non-vision systems (individual vehicle control through the reading of the text of the vehicle license plate) and in particular the advantage over visual systems as the infrastructure installation cost is much lower, since it does not require of a wiring system. In addition, the text recognition process of the plate is carried out in the SPIN-V device then only the text is upload to the cloud instead of an image or video. In summary, the SEI-UVM allows distributing the reserve management to the users and it is able to gather more information and communication with the user. Finally, the OBNiSE provides the management of the parking spaces status to be consumed by the mobile app.

This implementation gave way to the implementation of concepts of a smart city on campus, towards the construction of a Smart Campus, which would allow evaluating strategies in a system with the same complexity of a big city, but in a environment of greater control, before implementing it in a traditional city or a smart city. This proposal is framed as part of the university’s strategy to support the government’s interest in transforming the city of Guadalajara as the first smart city in Mexico [18].

As a result, future work will be focused on establishing the replication strategy in a smart city such as Guadalajara and assessing the scalability of the proposal, validating the scalability and interoperability properties, key elements necessary for the implementation of solutions in a smart city [19,20]. In addition, work will be done on the development of intelligent prediction algorithms to be implemented as a Software as a Service (SaaS) in OBNiSE, to be consumed by the mobile app, and optimize search results and services for the user.

## Abbreviations

The following abbreviations are used in this manuscript.

ACO	Ant Colony Optimization
AFOV	Angular Field of View
API	Application Program Interface
CIIDETEC-UVM	Centro de Investigación, Innovación y Desarrollo Tecnológico, UVM
EAST	An efficient and Accurate Scene Text
FOV	Field of Vision
GPS	Global Positioning System
hFOV	Horizontal Field of View
hPPM	Horizontal Pixel Per Meter
IoT	Internet of Things
ICT	Information and communication Technology
LED	Light-Emitting Diode
OBNISE	Observatorio Nacional Digital de Smart Environments
OCR	Optical Character Recognition
PC	Depth of Field
RAM	Random Access Memory
Rasperri PI	Reduced Programmable computer
ROI	Regions of interest
SaaS	Software as a Service
SEI-UVM	Smart Parking System of Universidad del Valle de Mexico
SPIN-V	Smart Vehicular Presence Sensor
UVM	Universidad del Valle de México
vFOV	Vertical Field of View
vPPM	Vertical Pixel Per Meter
WD	Working Distance
